# Ascites as the Presenting Sign of Systemic Lupus Erythematosus

**DOI:** 10.7759/cureus.23231

**Published:** 2022-03-16

**Authors:** Samuel G Cook

**Affiliations:** 1 Internal Medicine, Wright State University Boonshoft School of Medicine, Dayton, USA

**Keywords:** lupus peritonitis, peritoneal serositis, serositis, ascites, systemic lupus erythematosus

## Abstract

Although systemic lupus erythematosus (SLE) can manifest differently in each patient, ascites is a rare first sign. The diagnosis of SLE can be easily missed when the initial presentation is uncommon. A 39-year-old male presented with painless abdominal fullness and was found to have ascites, thrombocytopenia, and anemia. He was initially diagnosed with Evan’s syndrome and treated with prednisone. Upon follow-up, he had worsening thrombocytopenia and was found to have a positive antinuclear antibody, anti-double-stranded DNA antibody, and low complement levels consistent with SLE. He was treated with methylprednisolone, intravenous immunoglobulin, and mycophenolate mofetil with improvement.

## Introduction

Systemic lupus erythematosus (SLE) can involve nearly every organ system and manifests differently in each patient. It is considered one of the “great imitators” in medicine, as the diagnosis can be easily missed when the initial presentation is uncommon. Only 6% of patients have serositis as their first sign of lupus and it is rare to have ascites as the initial manifestation of their disease [1]. This case follows a man with new-onset ascites whose underlying diagnosis of SLE was elusive at presentation.

## Case presentation

**Figure 1:**
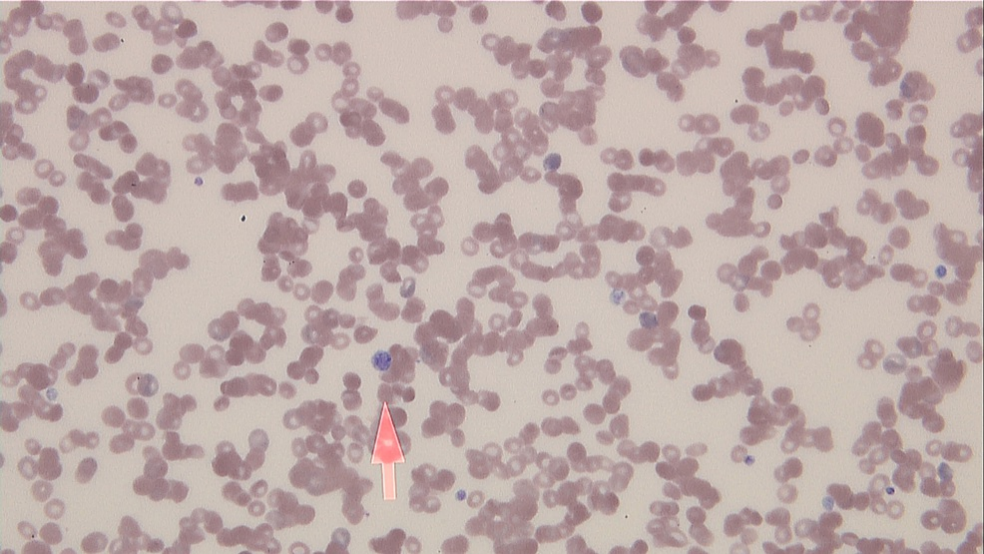
Peripheral blood smear. The peripheral blood smear shows giant platelets (arrow) and anisocytosis. Schistocytes and spherocytes were not found.

**Figure 2:**
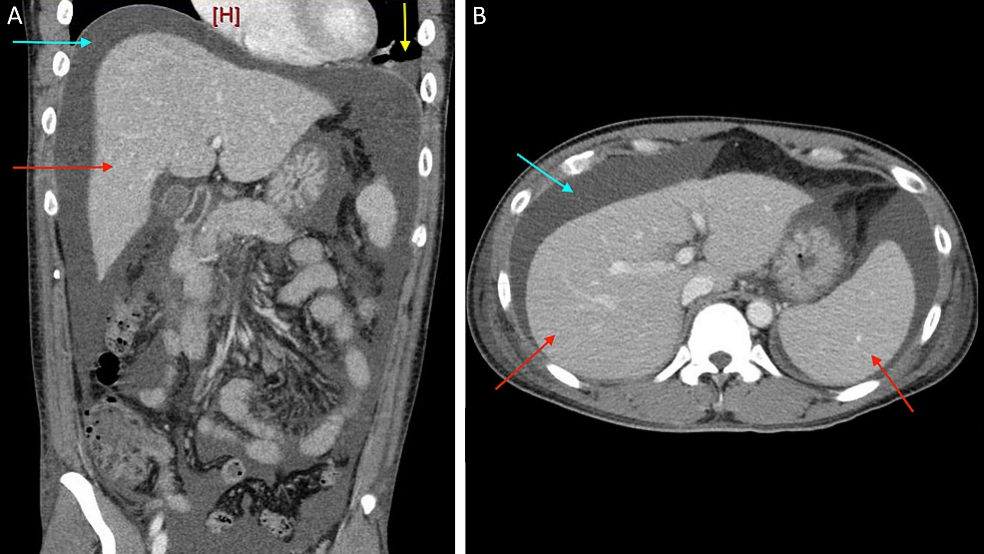
CT of the abdomen and pelvis. Coronal plane (A) and transverse plane (B) show a small pleural effusion (yellow), ascites (blue), and hepatosplenomegaly (red). No peritoneal lesions were identified.

A 39-year-old male with no past medical history was admitted with one week of painless abdominal fullness. He reported that his gums bled easily but otherwise denied additional symptoms. Physical exam was notable for abdominal distention without tenderness and palatal petechiae. Laboratory studies revealed thrombocytopenia (platelets: 27 K/μL), anemia (hemoglobin: 10.1 g/dL), elevated D-dimer (615 ng/mL), elevated fibrinogen (482 mg/dL), and hypoalbuminemia (albumin: 2.7 g/dL) without proteinuria. Peripheral smear showed giant platelets and no schistocytes (Figure 1). He had a positive Coombs test and positive warm agglutinin antibodies. Abdominal CT showed small bilateral pleural effusions, ascites, and hepatosplenomegaly (Figure 2). Abdominal ultrasound was negative for hepatic or portal vein thrombosis. The echocardiogram was unremarkable. Paracentesis was not performed due to thrombocytopenia.

The patient was initially diagnosed with Evan’s syndrome due to the presence of autoimmune hemolytic anemia and platelet destruction consistent with immune thrombocytopenia. He was started on prednisone 60 mg daily (body weight: 67 kg). His platelets subsequently improved to 43 K/μL and he was discharged home.

One week later, the patient was readmitted with worsening thrombocytopenia (platelets: 19 K/μL) despite medication compliance. Additional laboratory studies at that time were diagnostic of SLE with positive antinuclear antibody (ANA: 1:1280), positive anti-double-stranded DNA (anti-dsDNA: 1:40), and low complement levels (C3: 37 mg/dL; C4: undetectable). The remaining studies including rheumatoid factor (RF), anti-Ro/Sjögren's syndrome-related antigen A (SSA), anti-La/Sjögren's syndrome-related antigen B (SSB), anti-U1 ribonucleoprotein (RNP), and anti-Smith were negative. The patient also noted blurry vision; however, subsequent brain MRI and ophthalmology evaluation were unremarkable.

Therapy was initiated with IV methylprednisolone 125 mg every six hours and mycophenolate mofetil 500 mg twice daily. This regimen was chosen due to concern for severe disease with possible central nervous system involvement. Intravenous immunoglobulin (IVIG) 1 g/kg/day for two days was added for treatment of the hematologic manifestations. His thrombocytopenia improved and he was discharged home on oral prednisone and mycophenolate mofetil.

## Discussion

Ascites is seen in approximately 10% of SLE patients and can be caused by peritoneal serositis (lupus peritonitis), protein-losing enteropathy, nephrotic syndrome, and constrictive pericarditis [2]. Lupus peritonitis can present acutely with abdominal pain or chronically with a painless accumulation of ascites. The underlying physiology of lupus peritonitis is not fully understood. Ultimately, inflammation of the peritoneum leads to increased permeability and accumulation of exudative peritoneal fluid [3]. The ascites usually responds well to glucocorticoids [2,3].

Ascitic fluid in SLE has an expected serum-ascites albumin gradient (SAAG) of <1.1 g/dL, consistent with etiologies other than portal hypertension. Lupus peritonitis is considered a diagnosis of exclusion [3]. Constrictive pericarditis is evaluated with echocardiogram and nephrotic syndrome with 24-hour urine protein measurement. Protein-losing enteropathy is diagnosed by determining alpha-1 antitrypsin clearance from a 24-hour stool collection [4]. Given the lack of proteinuria in this patient, enteropathy was likely the cause of his hypoalbuminemia while the ascites may have been due to enteropathy or lupus peritonitis.

The most common manifestations of SLE are fatigue (91.8% of patients), arthralgias (90.2%), fever (88.5%), oral ulcers or alopecia (86.9%), and malar rash (83.6%) [5]. Notably, this patient did not have any of these symptoms. The 2019 European League Against Rheumatism (EULAR)/American College of Rheumatology (ACR) classification criteria of SLE specifically include serosal manifestations such as pleural and pericardial effusions but do not mention peritoneal serositis [6]. Despite this, the patient still met the 10-point requirement for the diagnosis of SLE with a total score of 19 points.

## Conclusions

SLE rarely presents with ascites but should be considered in the differential diagnosis of patients with multisystem abnormalities. Clinicians should keep in mind that serositis in SLE can manifest with features not explicitly mentioned in the classification criteria.
